# DNA methylation evidence against the accelerated aging hypothesis of schizophrenia

**DOI:** 10.1038/s41537-017-0017-5

**Published:** 2017-03-23

**Authors:** Brandon C. McKinney, Huang Lin, Ying Ding, David A. Lewis, Robert A. Sweet

**Affiliations:** 1 Departments of Psychiatry, University of Pittsburgh, Pittsburgh, PA USA; 2 Departments of Biostatistics, University of Pittsburgh, Pittsburgh, PA USA; 3 Mental Illness Research, Education, and Clinical Center, VA Pittsburgh Healthcare System, Pittsburgh, PA USA

## Abstract

The accelerated aging hypothesis of schizophrenia posits that physiological changes throughout the body that are associated with normal aging occur at an earlier age in individuals with schizophrenia. Testing this hypothesis has been limited by problems measuring biological age. Recently, a method using DNA methylation levels at 353 genomic sites to produce “DNA methylation age”, an estimate of tissue biological age, was described and validated. We used this method to test the hypothesis in the postmortem superior temporal gyrus of 22 non-psychiatric control and 22 schizophrenia subjects. DNA methylation age correlated with chronological age in both non-psychiatric control (*r* = 0.95, *p* < 0.0001) and schizophrenia subjects (*r* = 0.96, *p* < 0.0001). Age acceleration did not differ between non-psychiatric control and schizophrenia subjects (*t* = 1.27, *p* = 0.21). Our findings suggest there is no acceleration of brain aging in schizophrenia. Larger studies using samples from multiple brain regions and homogenous cell populations will be necessary to confirm these findings.

## Introduction

The accelerated aging hypothesis of schizophrenia (SZ) posits that physiological changes throughout the body that are associated with normal aging occur at an earlier age in individuals with SZ.^1^ Indeed, SZ is associated with premature age-related physiological changes (e.g., insulin resistance, hyperlipidemia, decreased testosterone, osteopenia, skin thinning/wrinkling, sarcopenia, dendritic spine loss, cerebral cortical atrophy, and cognitive decline), and individuals with SZ die ~ 20 years prematurely.^1^ Testing this hypothesis has been limited by problems measuring biological age. Recently, a method using DNA methylation (DNAm) levels at 353 genomic sites to produce “DNAm age”, an estimate of tissue biological age, was described and validated.^2^ We used this method to test the accelerated aging hypothesis of SZ in the postmortem superior temporal gyrus (STG).

## Results

**Fig. 1:**
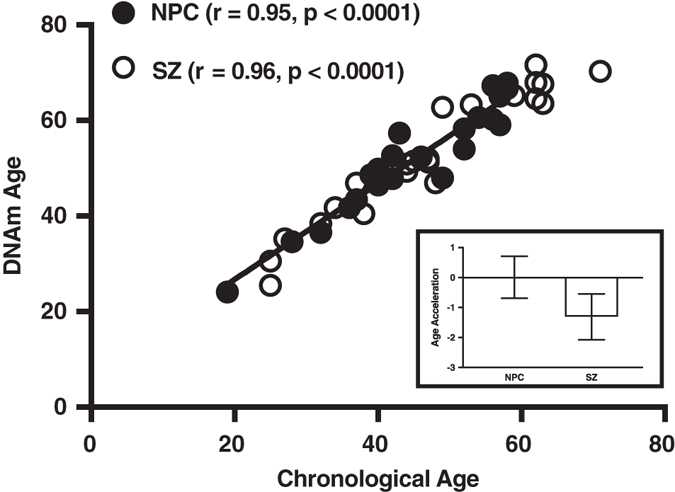
DNAm age analysis of the STG. (Main) Scatter plot of DNAm age vs. chronological age. *Filled circles* correspond to NPC subjects, *unfilled circles* to SZ subjects. The regression line of DNAm age on chronological age is shown in *black*. (Inset) Bar graph of age acceleration in and SZ subjects. The *bars* represent mean ± standard error of the mean. A negative value for age acceleration means the subject’s observed DNAm age is younger than the predicted DNAm age for an NPC subject of the same chronologic age. The average age acceleration in NPC subjects is 0 by definition

DNAm age was calculated as described in Horvath et al.^2^ DNAm age correlated with chronological age in both non-psychiatric control (NPC) (*r* = 0.95, *p* < 0.0001) and SZ subjects (*r* = 0.96, *p* < 0.0001) (Fig. 1). A linear model was built that regressed DNAm age on chronological age in NPC subjects (*black line* in Fig. 1). Age acceleration for each subject (NPC or SZ) was calculated as the corresponding residual resulting from the regression model. We found that age acceleration did not differ between NPC and SZ groups (*t* = 1.27, *p* = 0.21) (Fig. 1, inset). Dendritic spine density (DSD) was previously quantified for 17 of the 22 subjects in each group, and DSD was significantly reduced in the SZ group.^3^ However, age acceleration did not correlate with DSD in either the NPC (*r* = 0.23, *p* = 0.36) or the SZ (*r* = −0.27, *p* = 0.30) groups.

## Discussion

To our knowledge, this is the first study to use a DNAm-based approach in brain tissue to test the accelerated aging hypothesis of SZ. We did not find age acceleration in the STG of SZ subjects despite prior demonstrations of age-accelerated phenotypes (e.g., dendritic spine loss) in this brain region, including within these subjects.^3^ Aging in individuals with SZ may be accelerated in other brain regions. Using the same DNAm-based approach we used, brains from individuals with Huntington’s disease were shown to undergo brain-region-specific age acceleration,^4^ and the cerebellum was found to age more slowly than other brain regions in a study of normal brain aging.^5^ Similarly, age acceleration may occur in some cell types and not others, which will require cell-type-specific DNAm quantification to evaluate. DNAm data from STG layer 3 pyramidal neurons will be of particular interest given that these are the neurons on which dendritic spine loss in SZ subjects is thought to occur.

An important potential caveat is the small sample size. The study design, *N* = 22 for both NPC and SZ groups, provides 50 and 83% power to reject the null hypothesis (∆ mean age acceleration = 0) at a significance level of *α* = 0.05 (one-sided) for medium (*d* = 0.5, where *d* is the standardized effect size) and large effect sizes (*d* = 0.8), respectively. Large effect sizes for age acceleration have been observed in postmortem brain tissue of subjects with other brain pathologies including Huntington’s disease.^4^ In addition, other molecular/cellular indices of brain aging, such as age-dependent gene expression^6^ or telomere length,^7^ were not assessed. Leukocyte telomere length has been investigated in multiple psychiatric disorders, including SZ. Notably, the results suggest that, if present in SZ, biological age acceleration is modest and not specific to SZ.^7^

Thus, the current study provides initial evidence against, but does not preclude, accelerated aging of the brain in SZ. Our findings are consistent with the hypothesis that the brain changes in SZ, such as dendritic spine loss, are a consequence of aberrant neurodevelopment.^8^ Additionally, these findings neither exclude nor support the possibility of peripheral age acceleration in SZ. Given the absence of peripheral DNAm-based studies testing the accelerated aging hypothesis of SZ, and given that the bulk of data supporting accelerated aging in SZ comes from studies of peripheral measures,^1, 7^ we suggest a revision of the hypothesis in which SZ is conceptualized as a segmental progeria with accelerated aging peripherally. An intriguing subsequent question is whether a peripheral acceleration of aging could result from neurodevelopmental brain pathologies.

## Methods

Gray matter from the STG of brains from 22 NPC subjects and 22 subjects with either SZ (*N* = 16) or schizoaffective disorder (together referred to SZ), were recovered and processed as described previously.^3^ Each NPC subject was matched with a SZ subject for sex, hemisphere, and as closely as possible for postmortem interval and age, and, as a result, groups did not differ with respect to these parameters.^9^ Cohort characteristics are shown in the Table 1. All procedures were approved by the University of Pittsburgh Committee for the Oversight of Research and Clinical Training Involving the Dead and the Institutional Review Board for Biomedical Research. DNA was isolated and bisulfite-converted. DNAm was measured at 485,577 sites using Infinium HumanMethylation450 Beadchip Array (Illumina, San Diego, CA, USA) as previously described.^10^

**Table 1:** Cohort characteristics

Group	NPC	SZ
Number	22	22
Sex	17 M, 5 F	17 M, 5 F
Race	16 W, 5 B, 1 O	16 W, 6 B
Age (Years)	45.14 ± 2.30 (range 19–59)	47.14 ± 2.91 (range 25–71)
PMI (Hours)	17.58 ± 1.39	18.23 ± 1.79

*Note*: Data for continuous variables are presented as group average ± SEM.

*DNAm* DNA methylation, *DSD* dendritic spine density, *NPC* non-psychiatric control, SZ schizophrenia, *F* female, *M* male, *PMI* postmortem interval, *W* white, *B* black, *O* Other (Asian Indian)
